# GATA6 and TBX3 gene expressions analysis of indirect inguinal hernia sacs in children

**DOI:** 10.1007/s00383-025-06065-z

**Published:** 2025-07-07

**Authors:** Oguz Kizilkaya, Mehmet Said Koprulu, Hakan Gurkan, Mustafa Inan

**Affiliations:** 1 Department of Pediatric Surgery, Faculty of Medicine, Trakya University, Edirne, Türkiye; 2 Department of Medical Genetics, Faculty of Medicine, Trakya University, Edirne, Türkiye

**Keywords:** Indirect inguinal hernia, GATA6, TBX3, Gene expression, Single nucleotide polymorphism

## Abstract

**Purpose:**

The aim of the study is to examine the expression levels of the GATA6 and TBX3 genes in hernia sacs from patients with indirect inguinal hernia (IIH) in the Trakya region, Türkiye and shed light on the etiology of this common surgical disease in childhood.

**Materials and methods:**

In this cross-sectional study, GATA6 and TBX3 gene expression and single nucleotide polymorphism analyses were conducted on tissue samples obtained from 20 boys with IIH (study group) and 20 circumcised children (control group) who were operated in Trakya University Hospital, Pediatric Surgery Clinic. The tissue samples were collected from IIH sacs and prepuces. RNA isolation from tissue and complementary DNA (cDNA) synthesis were performed in accordance with the protocols of the kits used. In the DNA sequence variants (DSVs) study, DNA isolation was performed in accordance with the protocol of the kit used Allelic discrimination was also performed for the GATA6 rs1416421760 and rs1040912117 and TBX3 rs968910973 DVSs. Mann–Whitney U test was used to statistically compare the outcomes.

**Results:**

The expression of GATA6 demonstrated a significant increase (*p* < 0.001), while TBX3 expression exhibited a significant decrease (*p* < 0.001) in the study group. In terms of genotype and allele frequencies of GATA6 rs1416421760, rs1040912117, and TBX3 rs968910973 DSVs, no statistically significant difference was found between the study and control groups.

**Conclusion:**

According to the results of the study, it can be asserted that dysfunctions in the GATA6 and TBX3-mediated stages of the apoptotic pathway may contribute to the development of IIH.

## Introduction

From an etiological perspective, indirect inguinal hernia (IIH) occurs when the processus vaginalis (PV) is not completely obliterated [1–3]. Interruption in the apoptotic process in smooth muscle cells will stop the PV from being obliterated, causing the tissue to remain patent and develop IIH [2–5]. In daily clinical practice, certain families are encountered in which IIH manifests across multiple generations, from grandfather to grandson. It is put forward that IIH may be an inherited condition with a complex multifactorial origin [6–10]. Moreover, one of the main pillars of this idea is that inguinal hernia has been linked to syndromes caused by connective tissue defects, including William’s syndrome, Ehlers**–**Danlos syndrome, and Marfan syndrome. Furthermore, numerous genes, including GATA6, TBX1, TBX3 and COL1A1, are thought to be involved in the etiology of IIH [7–10] It has also been demonstrated that the TBX3 and GATA6 genes play a role in the regulation of apoptotic pathways during fetal development, and it has been noted that IIH patients have single nucleotide polymorphisms in these genes [7, 9].

The GATA transcription factors are involved in numerous essential functions during differentiation and remodeling in various tissues. The GATA family also plays a pivotal role in regulating proliferation and apoptosis [7, 11, 12]. Li et al. [13] have demonstrated that GATA6 enhances fibroblast growth and extracellular matrix production in response to TGFβ1 through the Wnt/β-catenin signaling pathway, while also inhibiting apoptosis. Moreover, evidence suggests that GATA6 suppresses apoptosis and influences tissue differentiation and remodeling through signaling pathways, such as TGFβ1 and Wnt/β-catenin [13, 14]. Qiao et al. [7] have suggested that alterations in GATA6 expression may be linked to developmental disorders associated with IIH. In this context, considering programmed cell death of smooth muscle as a crucial mechanism guiding testis development and function, it can be considered that GATA6 may associated with the patency of PV. In the same study, it was hypothesized that altered expression levels resulting from DNA sequence variants (DSVs) in the GATA6 promoter regions could contribute to the persistence of PV in patients with IIH, reported two functional sequence variants (g.22168361C > A and g.22169106C > T) [7].

T-box transcription factors, specifically, TBX3 expression has been observed in the mesoderm of mammals and detected in various tissues, including the nervous system, skeleton, eye, heart, kidneys, lungs, pancreas, mammary glands, and notably the genital ridge during organogenesis [8, 9, 15]. Given that the testicle originates from the genital tubercle, it is essential to thoroughly investigate the role of TBX3 in the etiology of IIH. Furthermore, the specific genes and pathways through which TBX3 variants contribute to IIH remain to be elucidated. Zhao et al. [9] have proposed that TBX3 gene variants might be involved in IIH by reducing TBX3 levels, potentially affecting the formation of the inguinal ring and thereby influencing IIH development. If this study is examined in more detail, it is hypothesized that DSVs in the TBX3 promoter regions could mediate the persistence of the PV by altering TBX3 expression levels. A heterozygous deletion variant (g.4820_4821del) was identified in a patient with an IIH, and it is believed that this variant significantly reduces TBX3 promoter activity [9]. On the other hand, a comprehensive review of the literature suggests that TBX3 likely regulates apoptosis, tissue differentiation, and remodeling through signaling pathways, such as TGFβ1 and Wnt/β-catenin [16–18].

Apoptosis, tissue differentiation, and remodeling processes may play a role in the embryological development of both the PV and the prepuce [19–21]. Furthermore, it is plausible to infer that, although their embryological origins differ, the development of these two tissues regulate by similar genetic and molecular signaling pathways, such as TGF-β and Wnt [22–25]. In light of the aforementioned literature, we hypothesize that genetic alterations in GATA6 and TBX3, particularly within their promoter regions, may contribute to the failure of PV obliteration and the development of IIH. In this context, we aimed to investigate the expression levels of GATA6 and TBX3 in hernia sacs obtained from patients with IIH and in prepuce tissues from circumcised healthy boys in our study population. In addition, we think that two variants in the GATA6 promoter region (rs1416421760, MAF: A = 0.000023; rs1040912117, MAF: T = 0.000043) and one variant in the TBX3 promoter region (rs968910973, MAF: A = 0.00004), all with minor allele frequencies (MAF) of less than 1%, may play a regulatory role in GATA6 and TBX3 expression levels in patients with IIH. According to the best of our knowledge, the GATA6 and TBX3 genes have not been previously examined in human IIH tissues. This study may provide significant insights into the embryological development of the PV and prepuce, and contribute to elucidating the genetic and molecular basis of inguinal hernias in children.

## Materials and methods

Children aged 0 to 18 who underwent surgery for IIH or circumcision were included in the study at the pediatric surgery clinic of Trakya University Hospital between January 2020 and October 2020. Since the patients were under 18, their legal guardians provided their written informed consent. The study was approved by local ethics committee.

Patients who visited our outpatient clinic and underwent herniorrhaphy with a diagnosis of IIH were included in the patient group of the study, while those who were decided to undergo circumcision surgery were included in the control group. Children who had undergone abdominal or inguino-scrotal surgery and were diagnosed with genetic disorders, as well as those with micropenis, hydrocele, undescended testes, incarcerated or strangulated inguinal hernia, hypospadias, differences in sex development, or other genitourinary anomalies, as well as premature infants, were excluded from both groups in our study. Any of the patients in both group have not associated anomalies or diseases. The operations were performed by five different surgeons at the clinic, all of whom used the same standard surgical procedures. For each patient, 1 × 1 mm samples of the patent processus vaginalis (PPV) were excised in the course of IIH surgeries, employing the Herzfeld technique [26]. 1 × 1 mm prepuce tissue samples were obtained from healthy male children who were brought to our hospital for ritual circumcision, with the excision methods being dorsal slit, preputial clamping, or sleeve excision [27]. Using the proper venous puncture techniques, blood samples were drawn from both patient groups and placed into blood tubes containing EDTA. The RNA-free solution (Invitrogen by Thermo Scientific, Waltham, USA) was added to the excised tissue before being placed in an Eppendorf tube and delivered within an hour to the Trakya University Department of Medical Genetics. A power analysis was conducted indicating that a total of 40 cases, 20 from IIH and control groups, should be included in the study with a 5% margin of error and 80% power.

RNA isolation from tissue (PureLink™ RNA Mini Kit, Invitrogen by Thermo Scientific) and complementary DNA (cDNA) synthesis (High Capacity cDNA Reverse Transcription Kit, Invitrogen, Applied Biosystems, Thermo Scientific, Waltham, USA) were performed in accordance with the protocols of the kits used. Expression of TBX3 and GATA6 genes and ACTB gene as housekeeping gene were analyzed in Applied Biosystems Step One Plus™ Real-Time PCR (Applied Biosystem, San Mateo, USA) device using TaqMAN Gene expression assay (Thermo Scientific, Waltham, USA) kit. The real-time PCR gene expression protocol was as follows: hold at 50 °C for 2 min, 95 °C for 2 min, Denaturation: 95 °C for 1 s and Anneal-Extend: 60 °C for 20 s, 40 cycles.

Gene expression analysis was performed in triplicate for each case. The mean C_T_ values of each case studied in triplicate were calculated separately for each gene. In each case, ACTB housekeeping gene with different expression levels were subtracted from the C_T_ value and ΔCt values of the cases were determined for the relevant gene. ΔΔCT and 2^-ΔΔCT values of each case and each assay were calculated from the ΔC_T_ values obtained during the study using Step One Software 2.3.

In the single nucleotide polymorphism study, DNA isolation was performed in accordance with the protocol of the kit used (PureLink™ Genomic DNA Mini Kit, Invitrogen by Thermo Scientific, Waltham, USA). Genotyping was performed on an Applied Biosystems Step One Plus™ Real-Time PCR (Applied Biosystem, San Mateo, USA) device using the TaqMAN Assay™ (Thermo Scientific, Waltham, USA) kit. Detailed information about the in-house primer sequences designed for GATA6 rs1416421760, rs1040912117, and TBX3 rs968910973 DSVs is provided in Table 1.

**Table 1:** GATA6 rs1416421760, rs1040912117 and TBX3 rs968910973 primers

*GATA6* DNVs	Forward and reverse primers	Melting temperature
rs1416421760	ACGCTTTCTAGGAGACCGAC CTCGCTCCCTTTCGCTTACA	59,19 °C 60,11 °C
rs1040912117	CCCGAGATAGGGTCGGAGAA CCAGGCAGACAATGAGAGCC	60.18 °C 60.75 °C
***TBX3* DNVs**	**Forward and reverse primers**	**Melting temperature**
rs968910973	AGCGATCTCTCGATAAGCCAC CGCCTCTAGAATTCACCGGG	59,73 °C 60,25 °C

The real-time PCR DSVs protocol was as follows: pre-PCR Read: 60 °C for 30 s, 1 cycle, Holding Stage: 95 °C for 10 min, 1 cycle, Cycling Stage: 95 °C for 15 s and 60 °C for 1 min, 40 cycles, Post-PCR Read: 60 °C for 30 s, 1 cycle. Allelic discrimination was performed for the GATA6 rs1416421760 and rs1040912117 and TBX3 rs968910973 single nucleotide polymorphisms.

In our study, descriptive statistical methods were primarily used. The data were not normally distributed, and the Mann–Whitney *U* test was employed to statistically compare the outcomes. A p value of < 0.05 was considered statistically significant.

## Results

Twenty patients who were operated for IIH group and 20 healthy children who applied for circumcision and underwent circumcision operation were included as the control group. The mean age of the control group was 50.05 ± 40.47 months, and the mean age of the IIH group was 30.75 ± 35.17 months (Table 2). There was no statistically significant difference observed in the age values between the groups.

**Table 2:** Demographic data of patient and control groups

	Mean age ± SD (month)	Minimum age (month)	Maximum age (month)
Control group (n: 20)	50, 05 ± 40,47	4	120
IIH group (n: 20)	30,75 ± 35,17	1	120

IIH: Indirect inguinal hernia

**Fig. 1:**
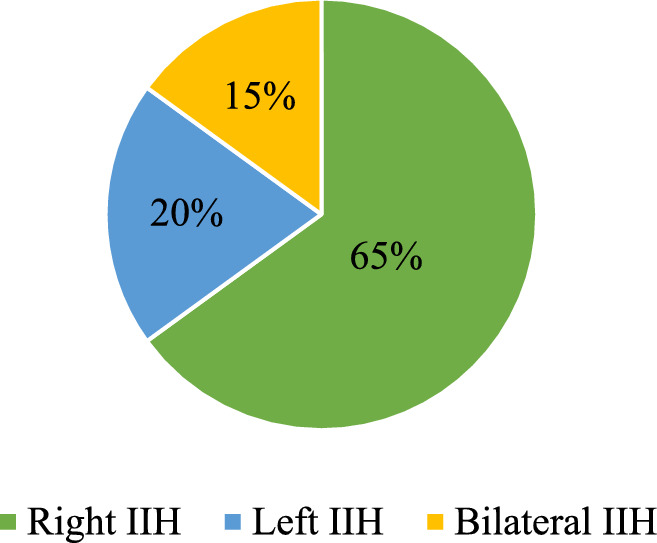
IH side distribution in patients

13 of the study's patients underwent surgery for right IIH (65%), 4 for left IIH (20%), and 3 for bilateral IIH (15%) (Fig. 1).

A statistically significant difference in GATA6 gene expression was found between the IIH group and the control group (p < 0.001). In addition, the IIH group had significantly lower levels of TBX3 gene expression as compared to the control group (p < 0.001) (Table 3).

**Table 3:** Gene expression results in tissues according to groups

	Groups	Number(n)	Mean ± SD	Min–Max (Median)	p* value
*TBX3*	IIH	20	0,12 ± 0,08	0,02–0,33 (0,1)	**0,001**
	Control	20	0,51 ± 0,29	0,08–1,3 (0,44)	
*GATA6*	IIH	20	94,14 ± 71,93	3,34–261,38 (63,81)	**0,001**
	Control	20	16,86 ± 42,49	0,65–194,01 (4,71)	

^*^; Mann–Whitney *U* test, p < 0.05 is highly statistically significant, IIH: Indirect inguinal hernia

No statistically significant difference was observed between the GATA6 and TBX3 expression levels in the right, left, and bilateral IIH patients when compared with each other (Table 4). DNA samples isolated from peripheral blood were used for genotyping and fluorogenic DSV analysis via real-time PCR. No statistically significant difference was found in the genotype and allele frequencies of GATA6 rs1416421760, rs1040912117, and TBX3 rs968910973 DSVs between the study and control groups.

**Table 4:** Comparison of gene expressions in right, left and bilateral IIH patients

	Right (n:13)	Left (n:4)	Bilateral (n:3)	p* value
*GATA6*	106,79 ± 80,92	81,56 ± 63,03	56,03 ± 18,84	0,713
*TBX3*	0,11 ± 0,69	0,17 ± 0,13	0,12 ± 0,08	0,704

^*^; Mann–Whitney U test, p < 0.05 is statistically significant

## Discussion

In our study, we found that TBX3 gene expression decreased, while GATA6 gene expression increased in PPV tissues in children with IIH. These findings suggest that the defect in PV closure might be caused by genetically linked disruptions in boys with IIH. In addition, in patient with IIH, the genotype and allele frequencies of GATA6 rs1416421760, rs1040912117, and TBX3 rs968910973 DSVs are not different from those in the normal population. Other DSVs or epigenetic modifications may contribute to this possible genetically derived disruption observed in patients with IIH.

In fact, there are numerous studies that elucidate the mechanisms of IIH formation in the literature. Environmental and genetic factors are frequently highlighted for this purpose, and the role of a multifactorial mechanism is widely acknowledged [1–6, 10]. The molecules and genes involved in these processes have been studied, and various data that may help clarify the etiology have been obtained. It has been proposed that any issues arising during both the sex-independent and sex-dependent periods of fetal life may result in IIH [28]. Some studies focused on using the peritoneum as a control tissue compared to PPV tissue to clarify the etiology of IIH. However, The PV is a distinct tissue with unique embryological development and histopathological features [2, 3, 29]. Furthermore, creating a control group for such a study poses significant ethical challenges. Despite the differences in the embryological origins of the PV and the foreskin, their developmental processes may have governed by overlapping genetic and molecular signaling pathways. In light of the literature review, we considered that the foreskin could be evaluated for comparison with PPV. The peritoneum is a structure covered by a layer of mesothelium on its surface and contains fibroblasts. In its deeper layers, it also contains vascular and nerve tissues, along with fibrous connective tissue. However, although the PV has peritoneal characteristics on its surface, it is a tissue that is surrounded by smooth muscle structures. It also has structural similarities with the Dartos muscle; therefore, PV may not be considered merely a simple peritoneal diverticulum [2]. An immunohistochemical study conducted in mice suggested that PPV does not have the same structural features as the peritoneum but has a structure similar to that of urogenital swelling. Consequently, it was stated that PV is not a diverticulum formed by intra-abdominal pressure but is associated with gubernacular structures at its origin [2, 3, 29]. On the other hand, it is not the PPV, but the obliterated PV that exhibits features similar to those of the peritoneum [2, 29]. Anatomically, the prepuce contains Dartos' fascia; however, histologically, the presence of smooth muscle fibers and its embryological development—originating from the urogenital swellings and may regulated by signaling pathways such as TGFβ1 and Wnt/β-catenin similar to those in the PPV—suggest that the prepuce may represent the tissue most closely resembling the hernia sac  [30–33]. Accordingly, to compare processes, such as embryological origins, smooth muscle content, and molecular pathways that drive differentiation and ECM remodeling within ethical boundaries, we chose to use preputial tissue instead of peritoneal tissues in the control group of healthy children.

Many theories in the literature address the etiology of testicular descent into the scrotum and the closure of the PV. According to Tanyel et al. [2, 3, 5, 29], the contraction of the smooth muscles surrounding the PV facilitates the descent of the testis into the scrotum. Apoptosis in smooth muscle cells causes the PV to obliterate after the testis descends into the scrotum. The PV remains open and is known as PPV, which predisposes to the development of IIH because the smooth muscle cells do not undergo apoptosis. PV is found in both sexes and PPV has the same morphological structure [3–7, 34]. Central catecholaminergic activity is involved in regulating peripheral sympathetic tone and gonadotropin-releasing hormone release. With the increase in sympathetic tone, the testicle descends. The apoptotic process is initiated by the dominance of parasympathetic tone, attributed to androgen receptor-dependent reduction in sympathetic tone within the smooth muscle cells of the PV, resulting in the obliteration of the PV [3]. The descent of the testis into the scrotum, as well as the formation and obliteration of the PV, occurs in a hormone-dependent manner. In a series of 46, XY patients with complete androgen insensitivity syndrome, external female phenotype, bilateral inguinal hernias, and bilateral abdominal undescended testis were reported [34].

Another theory is belonged to Hutson et al. [35, 36] that the gubernaculum is the main anatomical structure which controls the descent of the testicle and manages the migration process. According to this point of view, the genitofemoral nerve carries out mobilization of testicle from abdominal cavity to scrotum through Calcitonin Gene-Related Peptide (CGRP). In males, PV obliteration occurs after the descent of the testicle and any disruption in this process results in IIH. However, the authors put forwarded any comment about the formation and obliteration of the PV by the failed apoptosis of smooth muscle.

Numerous studies have investigated the impact of the genitofemoral nerve on inguinoscrotal pathologies [37–39]. Soyer et al. [37] suggested that CGRP is activated by electrophysiological stimulation of the genitofemoral nerve, thereby PV is obliterated as a result of the epithelial transformation. In addition, Mouravas et al. [38] found smooth muscle cells in hernia and hydrocele sacs. On the other hand, Li et al. [40] demonstrated the antiproliferative effect of CGRP in smooth muscle cell cultures and contributed to revealing the importance of the genitofemoral nerve in the obliteration processes of the PV. In the study conducted by Somuncu et al. [39], the authors introduce the idea of epithelial–mesenchymal transition (EMT) and support the theory of obliteration of PV.

One of the important transcriptional factors, GATA6 gene had studied by a Qiao et al. [7] in adult patients with IIH. The authors found two distinct functional sequence variants in the GATA6 gene promoter region linked to IIH [7]. It is also proposed that the increase in GATA6 expression directly enhances the synthesis of bone morphogenetic protein 2, thereby protecting the epiblast from apoptosis [41]. As a result, we concluded that the levels of GATA6 gene expression may be impacted by a different mutation in the promoter or exon regions. It has also been shown that GATA6 is necessary to maintain the differentiated state of smooth muscles [7, 11]. This suggests that the presence of smooth muscle in PPVs is due to increased GATA6 expression and supports our findings. On the other hand, GATA6 is a transcription factor that plays a critical role in apoptosis, tissue differentiation, and remodeling processes and Somuncu and Somuncu [42] reported that GATA6 variants may impair developmental signaling pathways required for PV smooth muscle differentiation or remodeling. In addition, previous studies have shown that apoptosis occurs with the reduction of GATA6 levels in the endoderm differentiation stage in the fetal life period [12]. By way of counterargument, Sun et al. [43] demonstrated in an epithelial cell culture study focused on apoptotic pathways that the increase in GATA6 expression mediated by the Angiotensin II receptor was associated with elevated Bax expression and induced apoptosis. However, another study described that Bax levels were low in PPV tissue due to the presence of smooth muscle [5]. Therefore, it may be considered that the effect observed with increased GATA6 expression levels in PPVs is not mediated through the Bax response.

In fact, numerous studies have explicitly indicated that the increase in TBX3 expression prevents apoptosis through various mechanisms [16, 44, 45]. In this context, a decrease in TBX3 expression levels could be expected to increase apoptosis. Although its antiapoptotic effect has been reported, partial suppression of TBX3 may not have the apoptosis-stimulating effect that was expected. According to Wensing and Campos [44], decreasing TBX3 expression may lead to the continuation of the antiapoptotic effect, depending on the qualities of the mRNA present in the environment. The factors leading to this result may be related with the specific subcellular localization of the mRNA, the secondary structure of the mRNA or the presence of RNA-associated proteins [44]. This evidence supports our findings, suggesting that the nonobliteration of PV is due to the absence of apoptosis since the decrease in TBX3 expression.

Wansleben et al. [46] and Krstic et al. [47] demonstrated in their separate studies that increased TBX3 expression in cancerous tissues is also associated with elevated levels of EMT. However, the inverse relationship between TBX3 and EMT was discussed by Somuncu and Somuncu [42], who indicated that a decrease in TBX3 expression induces a reduction in EMT, which in turn leads to PPV. Consistent with this, we believe that the reduction in TBX3 expression observed in the PPV tissues we studied could lead to decreased EMT levels and, consequently, to IIH.

TBX3 has been shown to support ureteral smooth muscle differentiation through Bone Morphogenic Protein 4 [48]. In a study conducted in mouse extraembryonic endoderm cells, a close relationship of TBX3 with the GATA6 promoter was found with a chromatin immunoprecipitation study and it was shown that GATA6 is a direct target of TBX3 [8]. However, in our study, we found decreased TBX3 expression despite increased GATA6 expression in PPVs, which contradicts this finding. It can be thought that this is because PPV originates from the epiblast-derived mesoderm. For this reason, we think that it would be appropriate to repeat this study in mesoderm/PPV tissues.

GATA6 and TBX3 have also been associated with some diseases encountered in childhood. GATA6 has been found to have altered expression levels not only in diaphragmatic hernia, type 1 diabetes mellitus, pancreatic agenesis, and growth and developmental delay but also in congenital heart diseases, particularly in conditions, such as Fallot's tetralogy, atrial septal defect, and persistent truncus arteriosus [49]. TBX3 has been mentioned in relation to ulnar mammary syndrome, pancreatic-related diseases, and cardiac pathologies [50]. Hutson et al. [51], in a review they authored in 2013, examined several congenital syndromes associated with undescended testes. In ulnar mammary syndrome, they proposed that the pathology responsible for the disruption in testicular descent is linked to dysfunctions in the Wnt/β-catenin signaling pathways attributable to a TBX3 gene mutation. This body of literature clearly indicates that changes in the expression levels of GATA6 and TBX3 may have significant implications for clinical practice.

In our study, we were not found difference in the genotype and allele frequencies of GATA6 rs1416421760, rs1040912117, and TBX3 rs968910973 DSVs among patients with IIH. This suggests that these specific DSVs do not regulate the expression levels of either gene. However, we believe that different DSVs located in the promoter regions of GATA6 and TBX3, or other epigenetic modifications, could potentially affect the expression levels of both genes.

There are some limitations of our study. The study performed COVID-19 era, the COVID-19 pandemic resulted in a small number of IIH and control cases, and the absence of patients with a family history in the IIH group is one of our study's limitations. We think that when assessing the study's findings, these circumstances need to be considered.

## Conclusion

Based on these findings from a small sample of patients, it was concluded that the pathways impacted by transcription factors like GATA6 and TBX3, which play a significant role in the mechanisms of smooth muscle cell dedifferentiation and apoptosis in the development of IIH, hold the key to understanding the etiology. These findings lead us to believe that the defect in the PV's ability to close may be genetically based, and that it may be caused by problems with the control of transcription in at least one of the TGF-β and WNT signaling pathways.

We also think that GATA6 and TBX3 gene sequence analyses should be carried out in IIH patients to understand the PV obliteration process at the molecular level and to clarify the associated apoptotic pathways, and that the functional effects of potential mutations in the promoter or exon regions should be determined in tissue culture or animal studies and larger patient series. In addition, we believe that performing genome analysis on families exhibiting a high incidence of IIH would be beneficial in comprehending the etiology of the disease.

## Data Availability

No datasets were generated or analysed during the current study.
